# Engineering a Bioactive PMMA–Silica Hybrid Scaffold for Enhanced Bone Regeneration

**DOI:** 10.1021/acsabm.5c02121

**Published:** 2026-03-03

**Authors:** Susaritha Ramanathan, Yu-Chien Lin, Huey-Yuan Wang, Ching-Li Tseng, Siwei Li, Wai-Ching Liu, Udesh Dhawan, Chih-Chien Hu, Ren-Jei Chung

**Affiliations:** ● Department of Chemical Engineering and Biotechnology, 34877 National Taipei University of Technology, Taipei 10608, Taiwan; ‡ School of Materials Science and Engineering, 54761 Nanyang Technological University, 50 Nanyang Avenue, Singapore 639798, Singapore; § Department of Stomatology, MacKay Memorial Hospital, Taipei 104217, Taiwan; ∥ Graduate Institute of Biomedical Materials and Tissue Engineering, College of Biomedical Engineering, 38032 Taipei Medical University, Taipei 11031, Taiwan; ⊥ International Ph. D. Program in Biomedical Engineering, College of Biomedical Engineering, Taipei Medical University, Taipei 11031, Taiwan; # Research Center of Biomedical Device, College of Biomedical Engineering, Taipei Medical University, Taipei 11031, Taiwan; ∇ International Ph. D. Program in Cell Therapy and Regenerative Medicine, College of Medicine, Taipei Medical University, Taipei 11031, Taiwan; ○ VSSAcademy Training & Education Ltd Office 6.072 sixth Floor, First Central 200, 2 Lakeside Drive, London NW10 7FQ, U.K.; ◆ Department of Food and Health Sciences, 435815 Technological and Higher Education Institute of Hong Kong, New Territories, Hong Kong 999077, China; ¶ Centre for the Cellular Microenvironment, Division of Biomedical Engineering, James Watt School of Engineering, Mazumdar-Shaw Advanced Research Centre, 3526 University of Glasgow, Glasgow G116EW, U.K.; ★ Bone and Joint Research Center, 38014 Chang Gung Memorial Hospital, Kweishan, Taoyuan 33305, Taiwan; ▲ Department of Orthopedic Surgery, Chang Gung Memorial Hospital, Kweishan, Taoyuan 33305, Taiwan; ▽ High-Value Biomaterials Research and Commercialization Center, National Taipei University of Technology (Taipei Tech), Taipei 10608, Taiwan

**Keywords:** poly(methyl methacrylate) (PMMA), bone regeneration, hybrid materials, osseointegration, biocompatibility

## Abstract

Bone health is crucial for maintaining mobility, structural integrity, and overall well-being. However, bone-related surgeries are the second most common type of tissue transplant worldwide. A reliable and bioactive material is needed to address these issues. This has led to growing interest in bone tissue engineering (BTE) as a viable substitute strategy. Poly­(methyl methacrylate) (PMMA) is a commonly utilized material in implant fixation and bone tissue applications due to its mechanical and chemical stability and ease of processing. However, its low bioactivity and poor osseointegration limit its effectiveness in bone repair, posing a significant technical challenge. In this study, we developed a PMMA–silica hybrid scaffold using tetraethyl orthosilicate (TEOS) as a silica source and 3-glycidoxypropyltrimethoxysilane (GPTMS) as a coupling agent. By incorporating silica, we aimed to enhance the bioactivity of PMMA, improve cell adhesion, and foster better integration with bone tissue. The effectiveness of the scaffold was assessed through *in vitro* tests for cell proliferation, adhesion, and differentiation, as well as through *in vivo* bone regeneration in a calvarial defect model using Sprague-Dawley (SD) rats over 12 weeks. The results demonstrated that the PMMA–silica hybrid scaffold effectively supports bone healing, highlighting its potential as a promising candidate for advanced bone regeneration therapies, combining mechanical strength with enhanced biological performance.

## Graphical abstract

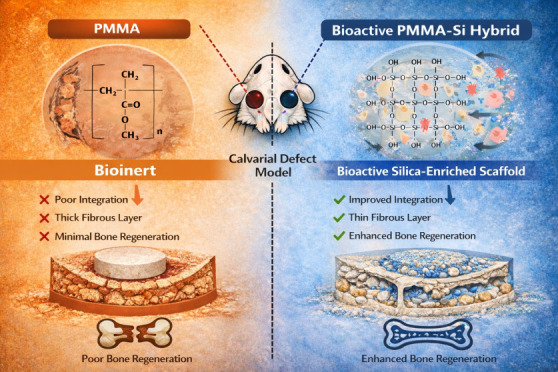

## 1 Introduction

Maintaining bone health is crucial for mobility, support, and protection of the body, while bones also serve as a reservoir for essential minerals.^1^ Bone transplantation is the second most frequently performed tissue transplant globally, following blood transfusion, with an estimated 4 million individuals globally requiring such procedures annually.^2^ Annually, around 500,000 patients in the United States and Europe alone require bone substitutes, contributing to a global economic burden of US$5.5 billion for bone fractures, with total annual costs for bone repair reaching US$17 billion.^3^ Bone-associated illnesses, often caused by inflammation, injury, trauma, diseases, aging, or genetic factors, often lead to considerable morbidity, negatively impacting health and quality of life.^4^

Autografting, regarded as the gold standard in bone repair, involves harvesting bone from one area of a patient’s body and relocating it to the site of the defect. While recognized for its effectiveness, autografts face constraints due to limited availability and potential donor site complications, including pain and morbidity. In contrast, allografts utilize decellularized bone from human donors, offering an alternative approach. However, they pose risks such as fracture, nonunion, infection, and immune responses.^5^ These challenges highlight the need for alternative solutions, making bone tissue engineering (BTE) a field of significant interest. Among the BTE approaches, scaffold-based strategy holds substantial promise in mitigating numerous constraints linked to traditional bone grafting methods, thus presenting considerable potential for advancing bone defect therapies in the future.^6^

PMMA, a synthetic polymer has gained significant interest in the field of BTE. It is commonly utilized as bone cement for implant fixation in total joint replacement surgeries, including hip and knee arthroplasty, and also functions effectively as a bone filler and substitute.^7,8^ Due to its biocompatibility, convenient handling, ease of processing, and cost-effectiveness, PMMA is commonly chosen as a scaffold material in BTE.^9^ However, PMMA has significant limitations that impact its performance as a bone substitute. Although it is biocompatible, PMMA lacks bioactivity, which means it does not actively promote bonding with bone tissue. This limited bioactivity and inadequate osseointegration result in a nonbonding interface between the implant and surrounding bone.^9,10^ The lack of bioactivity in PMMA can result in the development of a thick fibrous tissue layer between the implant and bone. This fibrous tissue acts as a barrier that further hinders direct integration with the bone, potentially leading to bone resorption, a process where the surrounding bone tissue is gradually broken down. Over time, these effects may result in implant loosening and, ultimately, premature implant failure, presenting significant challenges in ensuring long-term stability and success in BTE applications.^11^ Although naturally derived polymers such as collagen, gelatin, and chitosan possess intrinsic bioactivity, rapid degradation, and limited mechanical stability can restrict their use in applications requiring sustained structural support.^12^ Therefore, PMMA was intentionally selected in this study for its clinical relevance and mechanical reliability.^13,14^

Hybrid materials are the type of materials comprising organic and inorganic materials at submicron or nanoscale dimensions.^15^ Due to the molecular level interaction between their constituent compounds, hybrid materials are expected to yield novel properties, augmenting the inherent functions of their individual organic and inorganic constituents.^16^ This emerging class of materials combines diverse mechanical, chemical, and biological properties, resulting in high-performance materials characterized by an exceptional balance of strength, toughness, and tunable characteristics.^17,18^

In the context of bone regeneration, silica-based hybrid materials have attracted significant interest. Several studies have reported the use of tetraethyl orthosilicate (TEOS) as a silica precursor and 3-glycidoxypropyltrimethoxysilane (GPTMS) as a coupling or cross-linking agent to chemically integrate inorganic silica networks with organic polymers. For example, Gao et al. reported the synthesis of poly­(γ-glutamic acid)–silica hybrid nanofibrous scaffolds using TEOS and GPTMS as silica source and cross-linker for bone regeneration. The study demonstrated that increasing the TEOS concentration in the scaffold significantly improved the tensile strength, thermal stability, cellular proliferation *in vitro*, and alkaline phosphatase (ALP) activity.^19^ Similarly, Reyes-Peces et al., synthesized chitosan-silica hybrid aerogel using TEOS as an inorganic silica source and GPTMS as a cross-linker for bone regeneration. Their findings demonstrate that CS-GPTMS-silica hybrids exhibit excellent biocompatibility and bioactivity without cytotoxic effects, facilitating cell adhesion, cytoskeletal reorganization, and stress fiber formation via mature focal adhesion complexes.^20^ Importantly, *in vivo* bone-regeneration studies have already been reported for polymer–silica hybrid scaffolds fabricated using TEOS as the silica precursor and GPTMS as the cross-linking agent, demonstrating biocompatibility and enhanced bone formation in rat cranial defect model.^18^

In recent decades, silica-based materials have shown great promise in hard tissue engineering. Research has indicated that silicon enhances the bioactivity of biomaterials by stimulating type I collagen production, osteoblastic differentiation, and promoting repair of bone. In conditions such as osteoporosis and osteopenia, decreased osteoblast proliferation and activity have been linked to lower levels of biologically available silicon.^21^ In response, we synthesized a PMMA–silica hybrid scaffold specifically for BTE applications. Our main objective was to create a scaffold that combines the mechanical stability of PMMA with the bioactivity of silica to improve bone healing and osteointegration. The scaffold’s biological performance including cell proliferation, adhesion, and differentiation was assessed through *in vitro* cell studies. Additionally, *in vivo* bone regeneration was evaluated in a calvarial defect model using Sprague-Dawley (SD) rats over a 12-week period (Figure 1). Incorporating silica into PMMA scaffolds aims to address the limitations of PMMA by enhancing bioactivity and supporting bone integration, providing a dual advantage of mechanical support and active interaction with healing bone tissue. Overall, the silica integration within hybrid scaffolds offers a promising approach to developing materials that not only provide structural integrity but also actively facilitate bone regeneration and repair. The novelty of this study lies in demonstrating that a TEOS/GPTMS-derived silica phase can be integrated into a mechanically stable PMMA scaffold, thereby transforming an otherwise bioinert PMMA matrix into a bioactive, osteogenic platform, resulting in enhanced bone regeneration, as validated by both *in vitro* bioactivity/osteogenic assays and *in vivo* rat calvarial defect model.

**Figure 1:**
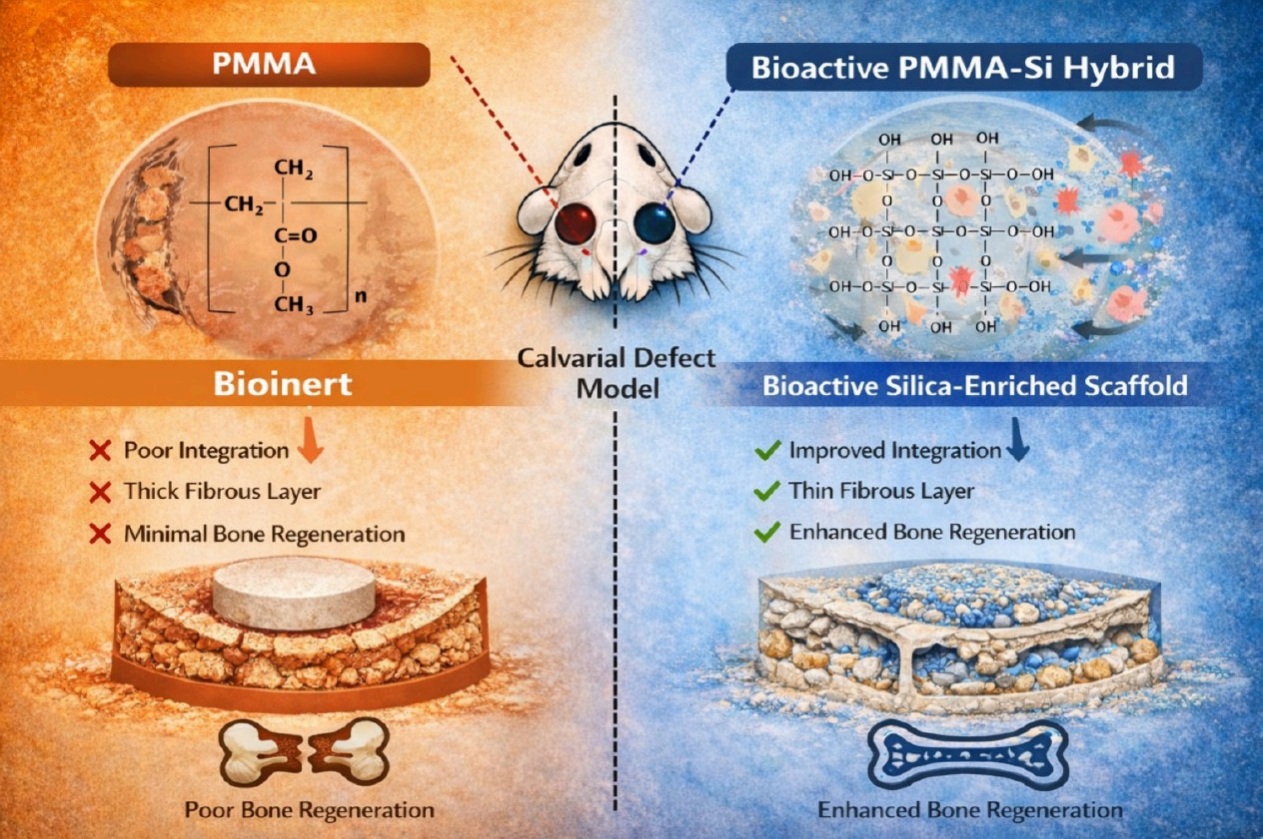
Comparison of PMMA and PMMA–Si hybrid scaffolds in calvarial defect healing in a Sprague-Dawley (SD) rat model. The left panel highlights the bioinert characteristics of pure PMMA, which lead to poor integration with host tissue, thick fibrous encapsulation, and minimal bone regeneration. In contrast, the right panel shows the PMMA–Si hybrid scaffold with a bioactive surface enriched in silanol (Si–OH) groups, facilitating enhanced cellular interactions, improved scaffold–tissue integration, a thinner fibrous layer, and superior bone regeneration.

## 2 Materials and Methods

### 2.1 Materials

Methyl methacrylate (MMA) was purchased from Showa (Japan). Azobis­(isobutyronitrile) (AIBN) and the Cell Counting Kit-8 (CCK-8) were obtained from Sigma-Aldrich (USA). 3-Glycidoxypropyltrimethoxysilane (GPTMS), tetraethyl orthosilicate (TEOS), and hydrochloric acid (HCl) were supplied by Jingming Chemicals (Taiwan). Cell culture media, including Minimum Essential Medium (α-MEM) and Dulbecco’s Modified Eagle Medium (DMEM), were purchased from Invitrogen (USA). MEM nonessential amino acids, 0.25% Trypsin–EDTA, and penicillin–streptomycin were obtained from Gibco (USA). Fetal bovine serum (FBS) was purchased from Biological Industries (Israel).

### 2.2 Synthesis of PMMA–Silica Hybrid Scaffold

The organic–inorganic hybrid scaffold was synthesized by tuning the ratio of PMMA to TEOS/GPTMS. To investigate the effects of different compositions, three varying concentrations of PMMA–silica hybrid scaffolds were prepared: 70% PMMA–Si, 60% PMMA–Si, and 50% PMMA–Si, along with a pure 100% PMMA scaffold as a control. The specific quantities of the reagents employed in the synthesis are detailed in Table 1. The synthesis process began by mixing TEOS and GPTMS in equal ratios. To facilitate hydrolysis, 2 mL of ethanol and 1 mL of 1 M HCl were added as catalyst. The resulting mixture was stirred for 2 h to ensure thorough hydrolysis and condensation reactions. After the initial hydrolysis step, MMA was added to the solution, along with 0.01 g AIBN as a free-radical initiator for the polymerization process. This mixture was stirred for 24 h at room temperature to allow for complete polymerization and uniform distribution of the silica network within the organic phase. The resulting homogeneous solution was carefully poured into custom molds and dried in an oven at 50 °C for 24 h to form a solidified hybrid scaffold structure.

**Table 1:** Specific Quantities of Reagents Used in the Synthesis of the Organic–Inorganic Hybrid Scaffold

composition	100% PMMA	70% PMMA–Si	60% PMMA–Si	50% PMMA–Si
MMA	10 mL	7 mL	6 mL	5 mL
TEOS	–	1.5 mL	2 mL	2.5 mL
GPTMS	–	1.5 mL	2 mL	2.5 mL
AIBN	0.01 g	0.01 g	0.01 g	0.01 g
Ethanol	–	2 mL	2 mL	2 mL
1 M Hcl	–	1 mL	1 mL	1 mL

Prior to biological experiments, PMMA and PMMA–silica samples were sterilized by immersion in 70% ethanol for 20 min, washed thoroughly with sterile PBS to remove residual ethanol, and exposed to UV irradiation for 30 min per side under aseptic conditions.

### 2.3 Characterization

To investigate the functional groups and molecular interactions within the scaffold, the samples were cut into 1 mm × 1 mm sections. These sections were then analyzed using attenuated total reflection-Fourier transform infrared spectroscopy (ATR-FTIR), allowing for detailed identification of the chemical bonds and molecular structures present within the material. The surface and detailed internal features of the scaffold were examined through scanning electron microscopy (SEM), which offered high-resolution images of its morphology and microstructure. The elemental composition was determined using energy-dispersive X-ray spectroscopy (EDX) using the S-3000H model from Hitachi, Japan. To improve conductivity, the samples were first sputter-coated with a thin layer of gold (Au) before analysis. The mechanical properties were evaluated using a universal testing machine (UTM) from Junyan Precision Machinery Co., Ltd., Taiwan. For the compression test, cylindrical samples measuring 15 mm in diameter and 30 mm in height were placed between stainless steel platforms, and a compression load was applied at a constant rate of 0.5 mm/s to assess the scaffold’s strength and its ability to withstand pressure.

The inorganic–organic weight percentage (wt%) ratios were assessed using thermogravimetric analysis (TGA) with STA7300, Hitachi, Japan. The samples, ground into a fine powder, were placed in a crucible and heated up from 20 to 800 °C at a constant rate of 10 °C/min under a continuous airflow, enabling accurate evaluation of their thermal stability and compositional changes. The phase identification of the synthesized PMMA and their hybrid samples was conducted using Malvern PANalytical X-ray diffractometer, UK; X’Pert3 Powder. The diffraction pattern was recorded over a Bragg angle range of 2θ = 10–80°, offering insights into the material’s structural properties. To further investigate the surface wettability of pure PMMA and PMMA–silica hybrid samples, the contact angle was measured using the sessile drop method. A droplet of deionized (DI) water was placed on the cleaned surface of each specimen, and a high-speed camera from FTA1000 (First Ten Angstroms, UK) was used to capture images of the droplet, allowing precise measurement of the contact angle. The surface roughness of the samples was measured using Atomic force microscopy (AFM), providing a detailed analysis of the surface topography at the nanoscale. The nanoindentation of the samples was performed using a TI 700 Ubi nanoindenter (Hysitron, USA) equipped with a Berkovich pyramid tip. This technique allowed for precise measurement of nanomechanical properties, including hardness, stiffness, and surface modulus, by applying a controlled force to the surface of the films and recording the depth of indentation.

### 2.4 Cell Culture

For cell studies, mouse fibroblasts (L929) and rat bone marrow-derived mesenchymal stem cells (rBMSCs) were used, as these cell lines are relevant for investigating cellular interactions and responses in BTE. L929 cells were cultured in DMEM, while MC3T3-E1 cells and rBMSCs were maintained in α-MEM, with all media supplemented with 10% FBS and 1% penicillin–streptomycin. All cultures were maintained at 37 °C in a humidified incubator with 5% CO_2_. Media were refreshed every 2–3 days, and cell morphology and confluence were monitored regularly to ensure optimal growth conditions. Cells were detached with trypsin solution when they reached 70–80% confluence for further subculturing and experiments.

### 2.5 Cell Viability Studies

The cytotoxicity of L929 cells and rBMSCs on pure PMMA and PMMA–silica hybrid samples was assessed using the CCK-8 assay. The samples were cut into 1 cm × 1 cm pieces and placed in a 48-well plate. Each well was seeded with 1 × 10^5^ cells and incubated at 37 °C with 5% CO_2_ for 1, 3, and 7 days. At each time point, 10% of the well total volume of CCK-8 reagent was added to the culture medium, and the plates were incubated for 1–2 h. Absorbance was then measured at 450 nm using a microplate reader to obtain quantitative data on cell viability.

### 2.6 Cellular Attachment

The scaffolds were prepared as 1 cm × 1 cm sections and placed into 24-well plates. Cells were seeded onto each scaffold at a density of 1 × 10^5^ cells per well and incubated at 37 °C in a 5% CO_2_ atmosphere for 1 day to support cell attachment and proliferation. Before imaging, the cells were stained with calcein-AM to specifically label live cells. The cell distribution and viability on the scaffolds were then analyzed using confocal fluorescence microscopy.

### 2.7 Alkaline Phosphatase (ALP) Activity

The osteogenic potential of pure PMMA and PMMA–silica hybrid scaffolds was evaluated by measuring ALP activity. rBMSCs were seeded at a density of 1 × 10^4^ per well in a 24-well culture plate and incubated at 37 °C with 5% CO_2_. The osteogenic medium was prepared by adding 10 mM β-glycerophosphate, 0.2 mM ascorbic acid, and 0.1 mM dexamethasone to DMEM. The cells were cultured using osteogenic medium and incubated at 37 °C with 5% CO_2_ for 1, 3, 7, and 14 days. The medium was refreshed every 3 days during the incubation period. At each designated time point, the culture medium was aspirated, and the wells were rinsed with phosphate-buffered saline (PBS). For cell lysis, 500 μL of 0.2% Triton X-100 was added to each well and incubated at room temperature for 20 min. Following lysis, 200 μL of assay buffer, 5 μL of 0.2 M magnesium acetate, and 2 μL of 1 M pNpp solution were added to each well to assess ALP activity. Absorbance readings were taken at a wavelength of 405 nm at both 0 and 4 min to quantify ALP activity.

### 2.8 Animal Model and Surgical Procedure

The procedures involving animals were approved by the ethics committee of Chang Gung Memorial Hospital (Taoyuan City, Taiwan). All experiments were approved by the Institutional Animal Care and Use Committee (IACUC) under affidavit no. 2023112802. This approval covered both primary rBMSC isolation and *in vivo* experiments. SD rats (8 weeks old, weighing 250–300 g) were used in the *in vivo* animal studies. Animals were randomly assigned to experimental groups (*n* = 3 rats per group). An anesthetic solution was prepared by mixing Zoletil 50 and Rompun 20 in a 1:2 ratio. The solution was administered via intraperitoneal injection at a dosage of 0.1 mL per 100 g of rat body weight. For tissue response study, the dorsal fur of each rat was shaved, and the exposed skin was thoroughly cleaned and sterilized. To ensure proper hydration, 1 mL of 0.9% sodium chloride solution was administered subcutaneously. For scaffold implantation, two 8 mm incisions were made along the dorsal region. Pure PMMA and PMMA–silica hybrid scaffold were implanted separately into each incision site. The incisions were then closed using 6–0 monofilament polypropylene sutures, and a topical application of 2% transdermal bupivacaine was used to prevent infection at the surgical sites. After 3 weeks, tissue samples from the implantation sites were collected and fixed in formalin for histological analysis. Hematoxylin and eosin (H&E) staining was performed to evaluate the cellular response and tissue integration within the scaffold. The stained sections were then analyzed for signs of inflammation and fibrosis around the implanted scaffolds. For calvarial defects, two 5 mm circular defects were created on the parietal bones of each rat cranium under anesthesia using a dental drill, ensuring minimal damage to the surrounding tissue. Scaffolds or materials under investigation were then implanted into the defect sites, while untreated defects served as controls. The surgical area was sutured, and rats were monitored postoperatively for recovery.

### 2.9 Micro-CT Analysis

Micro-CT imaging was performed on rats at 1, 2, 4, 8, and 12 weeks post-surgery to assess the progression of bone regeneration. During each scanning session, the rats were anesthetized with inhalation anesthesia, such as isoflurane, to minimize motion artifacts. Micro-CT scans were used to visualize and quantify the percentage of new bone formation within the defect sites. The data were analyzed to compare the progression of new bone formation between the treated and control groups over the study period.

### 2.10 Histological Analysis

For histological evaluation, the harvested calvarial samples were fixed in 10% formalin for 24 h, decalcified in EDTA solution (e.g., 10% EDTA, pH 7.4) for 2 weeks, and embedded in paraffin. Serial sections (5 μm thick) were prepared and subjected to Hematoxylin and Eosin (H&E) staining and Masson’s trichrome (MT) staining. H&E staining was used to assess the general morphology and cellular response within the defect area. Sections were stained with hematoxylin for 5 min, followed by eosin for 2 min. Stained sections were observed under a light microscope, and images were captured for analysis. H&E staining allowed for the visualization of inflammatory cells, fibrous tissue, and new bone formation. MT staining was performed to evaluate collagen deposition and the formation of mature bone matrix. Sections were stained using a standard MT protocol, which included staining with Weigert’s iron hematoxylin, followed by Biebrich scarlet-acid fuchsin, and differentiation with phosphomolybdic–phosphotungstic acid. Collagen fibers appeared blue, and cellular components appeared red. Stained sections were examined under a light microscope, and images were analyzed to assess the extent of collagen deposition and bone maturation.

### 2.11 Statistical Analysis

All experiments were performed in triplicate, and the results are presented as mean values with standard deviations (SD). One-way analysis of variance (ANOVA) was used for statistical analysis to evaluate differences between groups. All statistical analyses were conducted using Origin 2018 software. A *p* value of ≤ 0.05 was considered statistically significant, with significance levels indicated as * for *p* ≤ 0.05, ** for *p* ≤ 0.01, and *** for *p* ≤ 0.001.

## 3 Results and Discussion

### 3.1 Scaffold Preparation and Characterization

Figure 2a shows the optical images of pure PMMA and PMMA–silica hybrid samples. The scaffolds exhibited a smooth, well-structured, and uniform white appearance. The synthesis begins with the hydrolysis of TEOS, leading to the formation of silanol (Si–OH) groups. These groups subsequently undergo condensation reactions, resulting in the formation of a three-dimensional silica (SiO_2_) network.^22^ Simultaneously, MMA undergoes free-radical polymerization in the presence of AIBN, yielding PMMA.^23^ GPTMS serves as a bifunctional coupling agent, facilitating interaction between the organic and inorganic phases. The epoxy group present in GPTMS undergoes nucleophilic ring-opening reactions with hydroxyl or amine groups, enabling covalent bonding within the polymer matrix. Concurrently, the methoxy silane groups of GPTMS hydrolyze and condense with silanol groups from TEOS, further promoting the integration of the silica network into the composite structure.^24,25^

**Figure 2:**
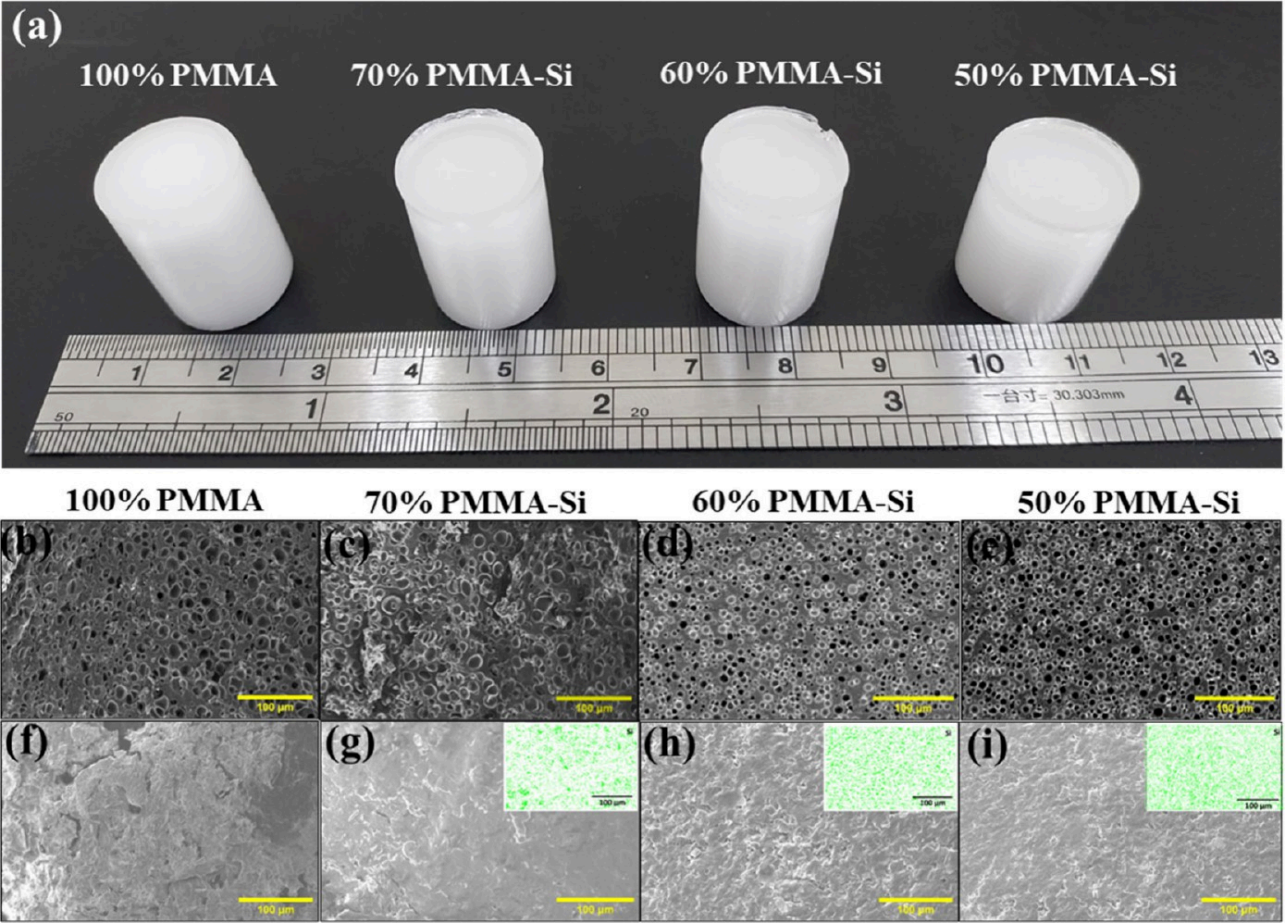
(a) Photograph of scaffolds composed of pure PMMA and different ratios of PMMA–silica hybrid. The samples shown include (from left to right) 100% PMMA, 70% PMMA–Si, 60% PMMA–Si, and 50% PMMA–Si. (b–i) SEM micrographs of horizontal cross sections (b–e) and vertical cross sections (f–i) of pure PMMA and PMMA–silica hybrids with various compositions (70% PMMA–Si, 60% PMMA–Si, 50% PMMA–Si). The insets in (g–i) show elemental mapping of silicon (Si), demonstrating its distribution across the samples. Scale bars: 100 μm.

Figure 2b–i shows SEM images of (b–e) horizontal cross sections and (f–i) vertical cross sections of pure PMMA and PMMA hybrid samples with different concentrations of silica. All samples, regardless of silica concentration, exhibited porous morphology. The average pore sizes of the samples were 18.8 ± 2.8, 18.6 ± 2.6, 17.855 ± 0.7, and 16.767 ± 1.0 μm for 100% PMMA, 70% PMMA–Si, 60% PMMA–Si, and 50% PMMA–Si, respectively. Moreover, the pore size ranges from 5 to 35 μm for all the samples. Previous research has revealed that microporosity in scaffolds significantly increases their specific surface area and permeability, creating additional sites for protein adsorption and tissue integration. This property enhances cellular interactions, as cells can adsorb more osteogenic-related proteins through membrane receptors, thereby improving osteogenic functions, such as attachment, proliferation, differentiation, and biomineralization. Moreover, the capillary forces generated by microporosity enhance the adhesion and migration of bone-related cells across the scaffold surface, allowing for effective cell penetration even through smaller pores.^26,27^ The insets in Figure 2g–i display the elemental mapping of silicon, confirming that silica is uniformly distributed throughout the scaffold. The average elemental composition of the scaffolds was analyzed using EDX, with detailed results presented in Table 2. The analysis confirmed that all hybrid scaffolds, except the pure PMMA sample, showed the presence of silica. The silicon content was measured at 7.7 ± 0.9%, 12.44 ± 1.61%, and 14.03 ± 2.03% for 70% PMMA–Si, 60% PMMA–Si, and 50% PMMA–Si scaffolds, respectively, demonstrating a clear correlation between the silicon weight percentage and the concentration of the silica precursor added.

**Table 2:** Average Weight Percentages of Carbon, Oxygen, and Silicon in Different Scaffolds (100% PMMA, 70% PMMA–Si, 60% PMMA–Si and 50% PMMA–Si) as Determined by EDX Analysis^a^

	weight percentage (%)		
scaffold	carbon	oxygen	silicon
100% PMMA	51.73 ± 3.96	48.26 ± 3.96	–
70% PMMA–Si	51.03 ± 1.33	41.26 ± 1.66	7.7 ± 0.9
60% PMMA–Si	41.97 ± 1.52	44.07 ± 1.02	12.44 ± 1.61
50% PMMA–Si	42.87 ± 1.09	41.42 ± 1.10	14.03 ± 2.03

a Values represent the mean ± SD calculated from EDX spectra acquired from *n* = 3 different regions of each sample.

Figure 3a shows the XRD spectrum of pure PMMA and PMMA–silica hybrid samples with varying concentrations of silica. Pure PMMA exhibits an amorphous structure with three major broad bands.^28,29^ However, after the addition of silica, the intensity of these peaks was reduced in the hybrid samples. This suggests that the reduction in the XRD intensity is possibly due to the interaction between silica and PMMA.^30^ The absence of sharp peaks in pure PMMA and hybrid samples confirms the amorphous nature of both PMMA and silica.^31–33^ Figure 3b displays the FTIR spectrum of pure PMMA and PMMA–silica hybrid samples with different concentrations of silica. The peaks at 2927 and 1724 cm^–1^ are due to −CH stretching and carbonyl group (C=O) stretching vibrations. The peak at 1436 cm^–1^ is attributed to C–H deformation. Because of O–CH_3_ from the ester group, there is a peak at 1150 cm^–1^.^34–36^ Moreover, all three hybrid samples contain peaks at 791 and 1074 cm^–1^ due to the presence of Si–O–Si and at 963 cm^–1^ due to Si–OH.^37,38^ The FTIR results confirm the presence of both polymer and silica groups in the samples.

**Figure 3:**
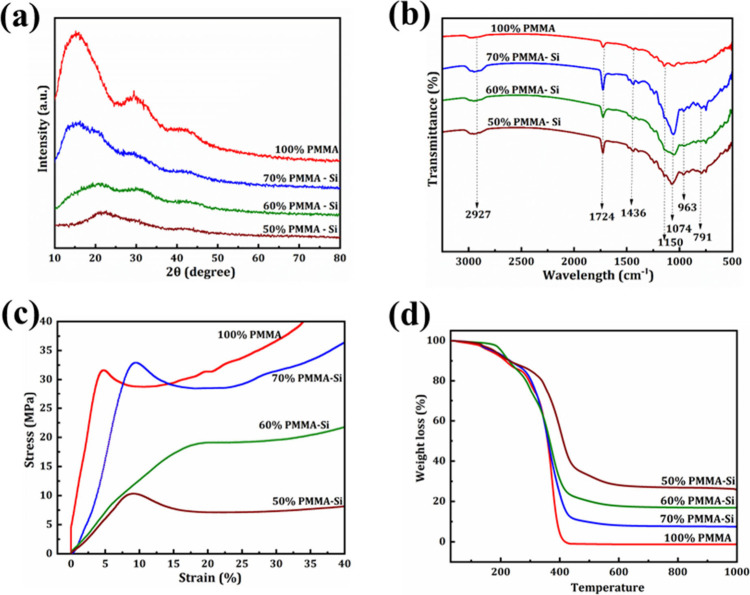
(a) XRD patterns and (b) FTIR spectra of pure PMMA and PMMA–silica hybrid scaffolds with various concentrations. (c) Compressive stress–strain curves of pure PMMA and PMMA–silica hybrid scaffolds. (d) TGA analysis of pure PMMA and PMMA–silica hybrid scaffolds.

The compressive stress–strain curves of pure PMMA and PMMA hybrid samples with different concentrations of silica (Figure 3c) exhibit a characteristic shape that is consistent with findings from other researchers.^39^ The Young’s modulus and the ultimate stress of the pure PMMA scaffold were 535.5 ± 4.1 and 31.5 ± 1.08 MPa, respectively. Beyond this ultimate stress peak, the stress decreases slightly and then gradually increases, suggesting that the material undergoes initial elastic response and then strain softening followed by strain hardening.^40^ Similar response was obtained for the PMMA hybrid samples. The Young’s moduli were 468.9 ± 14.6, 343.1 ± 9.5, and 115.7 ± 11 MPa and the peak stresses were 30.5 ± 2.1, 19.9 ± 1.2, and 11.7 ± 1.4 MPa for 70% PMMA–Si, 60% PMMA–Si, and 50% PMMA–Si, respectively. For the PMMA hybrid samples, the Young’s modulus and ultimate stress were reduced upon increasing the concentration of silica. This is probably due to the addition of TEOS and GPTMS. Previous studies have shown that when the TEOS dosage exceeds 20%, tensile strength decreases due to rapid hydrolysis, leading to rigid SiO_2_ structures, incomplete cross-linking, and agglomeration in the polymer solution, making the hybrids more brittle.^41^ Likewise, excessive GPTMS forms too many bridging bonds between inorganic and organic phases, increasing brittleness and reducing mechanical properties.^41^ The stress–strain curves demonstrate that all the scaffolds exhibit mechanical properties comparable to trabecular bone. Specifically, trabecular bone is known to have a Young’s modulus ranging from 50 to 500 MPa.^37^ These parameters indicate the stiffness and load-bearing capacity of the bone tissue. The fact that the scaffolds fall within this range suggests that they possess similar mechanical behavior to trabecular bone, making them potentially suitable for applications in BTE and orthopedic implants. This similarity in mechanical properties ensures that the scaffolds can provide adequate support and integration when used to repair or replace damaged bone.

Table 3 presents the results of nanoindentation analysis for pure PMMA and PMMA–silica hybrid samples. The nanoindentation results reveal a progressive reduction in surface-level mechanical properties with increasing silica content in the PMMA hybrid scaffolds. The surface modulus of pure PMMA is measured at 2.93 ± 0.32 GPa, which decreases significantly to 1.75 ± 0.73 GPa in the 50% PMMA–silica scaffold. Similarly, the hardness of pure PMMA, initially recorded at 0.29 ± 0.04 GPa, drops to 0.13 ± 0.089 GPa with 50% silica incorporation. Stiffness also shows a notable reduction, from 2.93 ± 0.63 μN/nm for pure PMMA to 1.99 ± 0.95 μN/nm in the 50% PMMA–Si scaffold. These declines in surface modulus, hardness, and stiffness reflect the reduced resistance to localized deformation, likely due to increased silica agglomeration and changes in the polymer matrix structure, including reduced polymer-chain continuity and packing uniformity near filler–polymer interfaces, increased local heterogeneity, and the formation of weak interfacial regions. These effects can reduce effective load transfer and increase localized compliance under indentation, thereby lowering resistance to deformation and diminishing surface mechanical integrity, consistent with the trends observed in the compression studies.^41^

**Table 3:** Nanoindentation Results Showing Surface Modulus, Hardness, and Stiffness of PMMA and PMMA–Silica Hybrid Scaffolds at Different Compositions

scaffold	surface modulus (GPa)	hardness (GPa)	stiffness (μN/nm)
100% PMMA	2.93 ± 0.32	0.29 ± 0.04	2.93 ± 0.63
70% PMMA–Si	2.60 ± 0.99	0.22 ± 0.001	2.84 ± 0.57
60% PMMA–Si	1.82 ± 0.63	0.19 ± 0.09	2.29 ± 0.91
50% PMMA–Si	1.75 ± 0.73	0.13 ± 0.09	1.98 ± 0.95

The TGA thermogram, which shows the weight loss percentage as a function of temperature for pure PMMA and PMMA–silica hybrid samples with varying concentrations of silica, is shown in Figure 3d. All of the samples exhibited a single-stage thermal degradation process.^42,43^ The weight loss begins at around 170 °C for all the samples, and the decomposition continues until 400–450 °C. Pure PMMA (100% PMMA) showed complete degradation, leaving no residue. However, the hybrid samples displayed residual silica after decomposition, with the amount increasing as the silica content increased. Specifically, the residual silica was found to be 7.69%, 16.92%, and 25.96% for 70% PMMA–Si, 60% PMMA–Si, and 50% PMMA–Si, respectively. Since TGA was performed in air, the residual mass at high temperature primarily reflects the bulk inorganic fraction (silica). In contrast, EDX provides local, surface-sensitive, semiquantitative elemental composition; therefore, direct quantitative comparison between TGA residue (bulk wt %) and EDX results is not expected.

Figure 4a–d shows the surface topographies of pure PMMA, 70% PMMA–Si, 60% PMMA–Si, and 50% PMMA–Si, as observed by AFM. The results suggest that pure PMMA shows a relatively smooth surface with an average roughness value of 55.35 ± 1.20 nm. However, for hybrid samples the roughness value increases with an increase in silica concentration, with roughness values of 85.75 ± 3.18, 96.36 ± 3.12, and 143.5 ± 3.53 nm for 70% PMMA–Si, 60% PMMA–Si, and 50% PMMA–Si, respectively, as shown in Figure 4e. Research has shown that surface morphology plays a critical role in the initial adhesion of osteoblasts. A rough scaffold surface tends to reduce fibroblast adhesion while enhancing osteoblast adhesion. Moreover, the increased surface area provided by a rough scaffold allows for greater osteoblast attachment, thereby accelerating the process of bone tissue repair.^44^ Figure 4f shows the wettability of pure PMMA and PMMA hybrid samples with different concentrations of silica. The average contact angles of 100% PMMA, 70% PMMA–Si, 60% PMMA–Si, and 50% PMMA–Si were found to be 65.98°, 57.45°, 53.84°, and 49.27°, respectively. From the results, it can be seen that all of the samples are hydrophilic in nature. However, for hybrid samples, with the addition of TEOS and GPTMS, the contact angle was further reduced, resulting in higher hydrophilicity. The enhanced hydrophilicity is due to the presence of oxygen-containing groups in the molecular structures of PMMA, TEOS, and GPTMS as well as surface silanol groups found on the surface of the inorganic phase.^45^ The majority of research has shown that hydrophilic surfaces provide a more favorable environment for facilitating cellular attachment.^46^ Studies have shown that cells exhibit optimal adhesion on surfaces characterized by contact angles ranging from 40° to 70°, implying that the observed hydrophilicity of the samples could be favorable for cell adhesion.^47^

**Figure 4:**
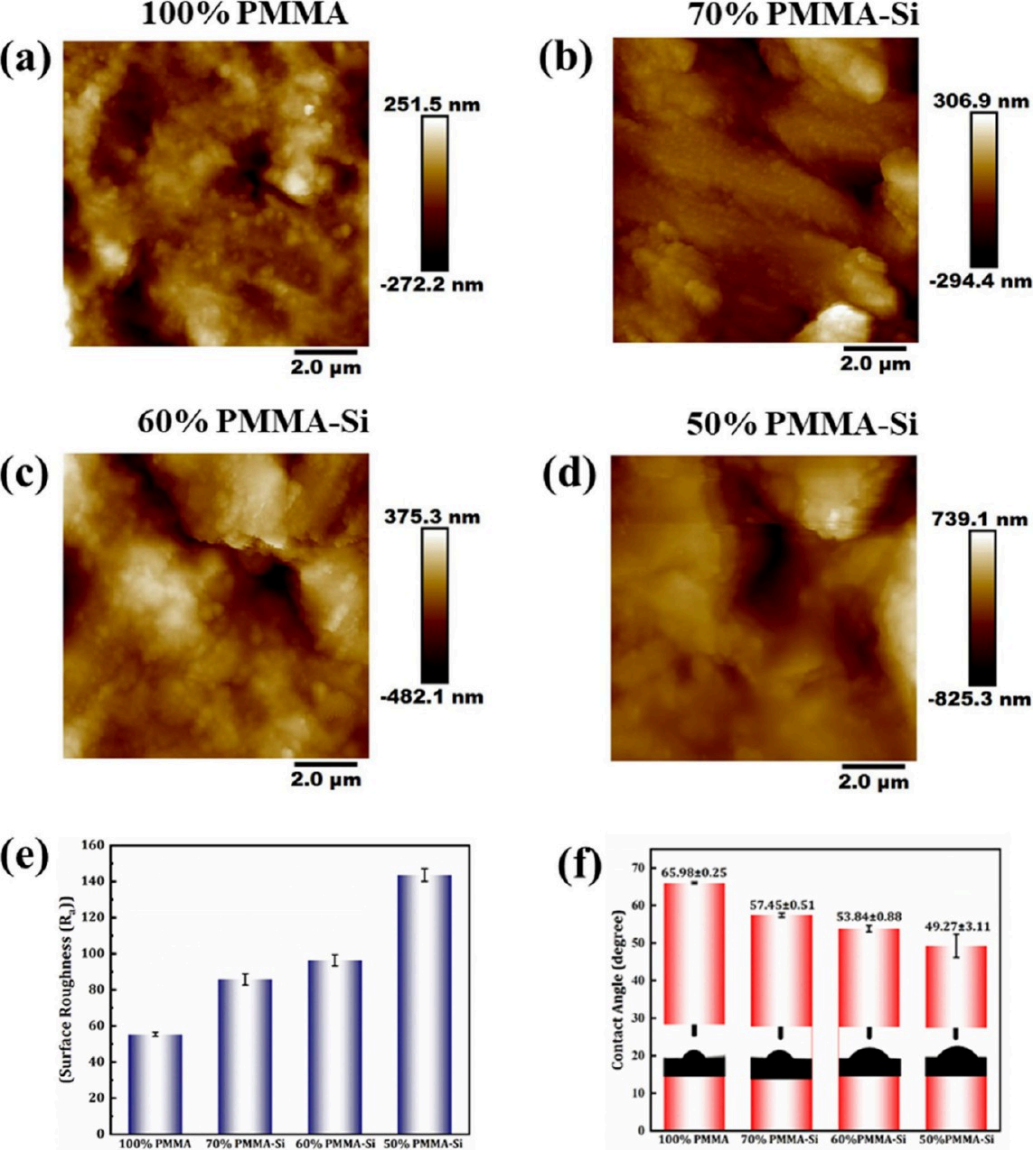
(a–d) AFM images of (a) 100% PMMA, (b) 70% PMMA–Si, (c) 60% PMMA–Si, and (d) 50% PMMA–Si with corresponding height profiles indicating surface roughness variations. (e) Average roughness (*R*_a_) values for the different compositions as measured from AFM. (f) Water contact angle measurements indicating the hydrophobicity of the surfaces for pure PMMA and PMMA–silica hybrid scaffolds.

### 3.2 *In Vitro* Cell Studies

Biocompatibility is the capacity of a biomaterial to perform effectively without inducing adverse biological reactions.^48^ An optimal scaffold must be nontoxic, immune to rejection, and capable of supporting cell attachment, migration, proliferation, and differentiation.^49^ Therefore, to evaluate the biocompatibility of pure PMMA and PMMA–silica hybrid samples, we used L929 and rBMSc cells, and the results are presented in Figure 5a,b. For L929 and rBMSc, the viability was more than 80% for all the samples. According to current ISO 10993–5 standards, cellular viability above 75% is considered safe, indicating that the material is nontoxic for use in medical devices.^50^ However, the percentage of cell viability for pure PMMA is lower when compared to hybrid samples. The reduced cell viability associated with PMMA is primarily due to its low bioactivity, which limits cell attachment, proliferation, and overall biocompatibility. Furthermore, incomplete polymerization of PMMA leaves residual monomers with cytotoxic properties. These unreacted monomers can leach into surrounding tissues, resulting in localized toxicity, inflammatory responses, and other adverse biological effects.^51^ Cell adhesion is essential in tissue regeneration, as it not only supports the structural integration of cells but also facilitates migration, survival, and guides differentiation and cell fate.^52^ The precise regulation of cell adhesion to biomaterial surfaces is critical for advancing tissue regenerative engineering. Therefore, we evaluated the adhesion of rBMSCs on the scaffold. The fluorescence images in Figure 5d illustrate the cell attachment and distribution on the surfaces of the pure PMMA and PMMA–silica hybrid scaffolds after 3 days of incubation. The images reveal that cell attachment and spreading are significantly enhanced on the PMMA–silica scaffolds compared to the pure PMMA scaffold, with an apparent increase in cell density and coverage as silica content increases. The 50% PMMA–Si scaffold, in particular, shows the most extensive cell coverage and spreading, indicating that higher silica content may provide a more favorable environment for cell adhesion. This enhanced cell distribution on PMMA–Si scaffolds could be attributed to the increased surface roughness and hydrophilicity introduced by the addition of silica. The ALP activity assay was performed to evaluate the osteogenic potential of various pure PMMA and PMMA–silica hybrid scaffolds, specifically 100% PMMA, 70% PMMA–Si, 60% PMMA–Si, and 50% PMMA–Si, over three-time intervals (Day 1, Day 7, and Day 14) (Figure 5c). ALP serves as an early marker of osteogenic differentiation, with its enzymatic activity indicating the progression of rBMSCs toward an osteoblastic phenotype.^53^ At Day 1, ALP activity remained low across all samples, including the control, indicating minimal early osteogenic differentiation. However, by Day 7, except for the control group, all other groups showed a marked increase in ALP activity, particularly in the PMMA–silica hybrid scaffold. This increase indicates that differentiation toward osteoblast lineage was initiated. Among the PMMA–Si compositions, the 50% PMMA–Si and 60% PMMA–Si groups exhibited notably higher ALP activity levels compared to both the 100% PMMA and 70% PMMA–Si groups, suggesting enhanced osteogenic potential as silicon content increased. By Day 14, this effect became more pronounced, with the 50% PMMA–Si group exhibiting the highest ALP activity, followed closely by the 60% PMMA–Si group. In contrast, both the 100% PMMA and control groups maintained significantly lower ALP levels, suggesting that the absence of silicon in the PMMA matrix diminishes its osteogenic stimulation capability. These observations align with existing studies that document the bioactivity of silicon in enhancing osteogenesis. Silicon is known to stimulate cellular responses that promote bone formation, which likely contributed to the enhanced ALP activity observed in silicon-containing PMMA hybrids.^54^ In this study, the 50% PMMA–Si composite, with its optimal silica concentration, elicited the strongest osteogenic response, as demonstrated by ALP activity levels. This indicates its suitability for applications in bone regeneration, providing a favorable scaffold environment for rBMSC differentiation. Based on the results from *in vitro* studies, the 50% PMMA–Si composite was identified as the optimal candidate for further investigation, due to its superior biocompatibility and significantly improved bioactivity relative to other formulations.

**Figure 5:**
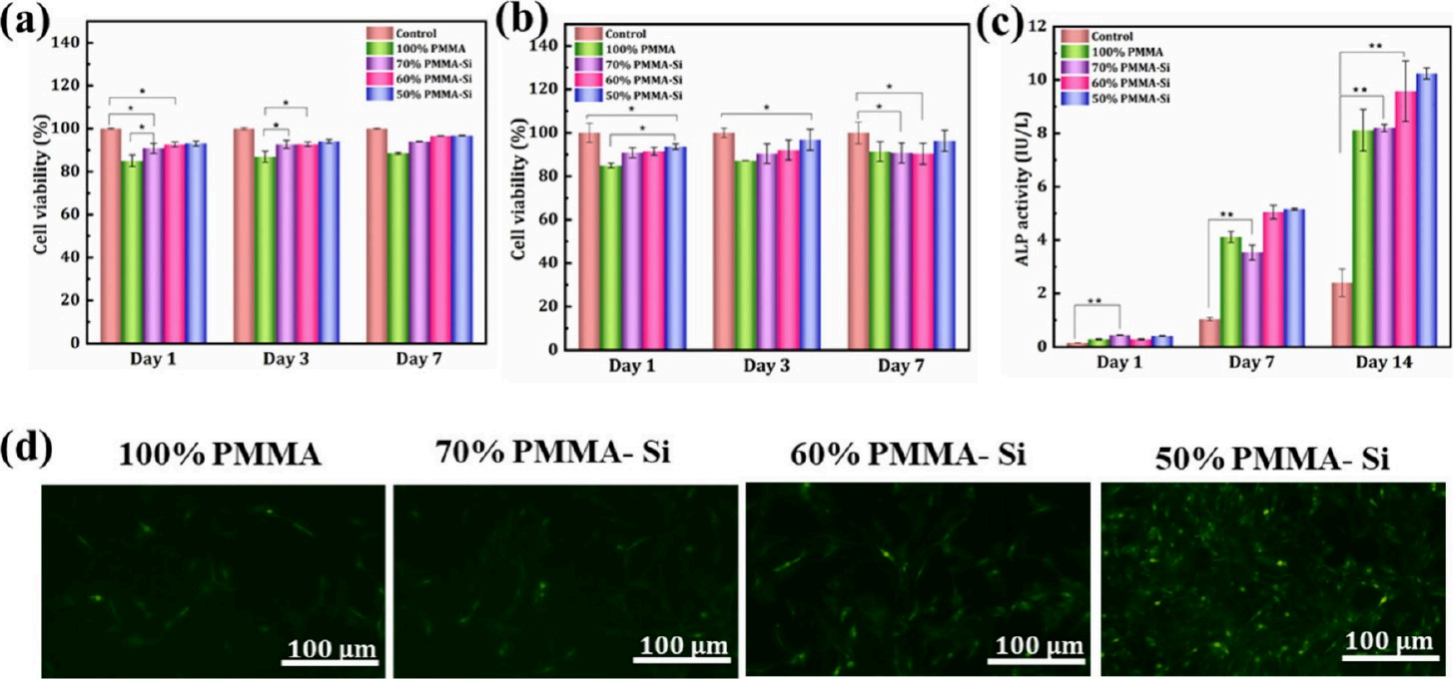
(a, b) CCK-8 assay results showing viabilities of (a) L929 cells and (b) rBMSCs on 100% PMMA, 70% PMMA–Si, 60% PMMA–Si, and 50% PMMA–Si over 1, 3, and 7 days, with significant differences marked. For each group *n* = 3; *p ≤ 0.05, **p ≤ 0.01, and ***p ≤ 0.001. (c) ALP activity in rBMSCs cultured on 100% PMMA, 70% PMMA–Si, 60% PMMA–Si, and 50% PMMA–Si over 1, 7, and 14 days. (d) Representative fluorescence images of rBMSCs stained with calcein-AM dye cultured on 100% PMMA, 70% PMMA–Si, 60% PMMA–Si, and 50% PMMA–Si substrates at day 3. Scale bars: 100 μm.

### 3.3 *In Vivo* Studies

This study aimed to evaluate the biocompatibility and degradation of scaffolds using H&E staining 21 days postimplantation in a rat model. The experimental groups included a control group, a 100% PMMA scaffold group, and a 50% PMMA–Si hybrid scaffold group, each assessed for their tissue response and scaffold degradation (Figure 6a–c). In the control group, the tissue appeared normal, showing well-preserved adipose tissue and normal dermal architecture. There was no evidence of an abnormal inflammatory response or excessive tissue reaction, indicating that the surgical procedure alone did not induce adverse effects. In both the 100% PMMA and 50% PMMA–Si groups, large central cavities were observed where the scaffolds had been implanted and subsequently removed after 21 days. These cavities indicate the former scaffold locations, and the surrounding tissue exhibited similar fibrotic responses. This encapsulation indicates that the host tissue reacted to both scaffold types by forming a fibrous capsule, a typical response to foreign materials.^55,56^ Importantly, no inflammation was observed in either scaffold group. There were no significant inflammatory cell infiltrations, such as macrophages or lymphocytes, suggesting that the acute inflammatory response had subsided by 21 days, and the tissue had transitioned into a healing phase characterized by fibrosis. This absence of inflammation indicates that both scaffold types were well-tolerated by the host tissue. In the 100% PMMA scaffold, there appears to be a distinct capsule formation with minimal or no integration between the scaffold and the surrounding tissue. The black arrows indicate gaps or clear boundaries, suggesting a possible foreign body response where the tissue is “walling off” the scaffold rather than incorporating with it. In contrast, the 50% PMMA–Si scaffold shows a more continuous tissue response. The asterisks highlight areas with improved integration, where the tissue appears to grow continuously across the scaffold. This suggests that the addition of silicon enhances biocompatibility, encouraging more natural tissue growth, resulting in less encapsulation and better integration with host tissue. The initial weight of the scaffolds prior to implantation and the final weight measured after 21 days of implantation remained unchanged. This consistency in weight suggests that no measurable degradation of the scaffold material occurred during the implantation period, indicating that both the 100% PMMA and hybrid PMMA with silica scaffolds maintained their structural integrity throughout the duration of the study. We further evaluated the tissue response in the calvarial defect area (Figure 6d,e). In 100% PMMA, the encapsulation layer surrounding the cavity is thicker compared to the 50% PMMA–Si. This indicates that pure PMMA causes a stronger reaction in the surrounding tissue, resulting in a denser encapsulation layer around the implant. But in 50% PMMA–Si, the encapsulation layer around the cavity is thinner, suggesting a milder response from the surrounding tissue. The addition of silica to the PMMA composite might be making the material more compatible with the tissue, reducing the reaction and leading to a thinner encapsulation layer.

**Figure 6:**
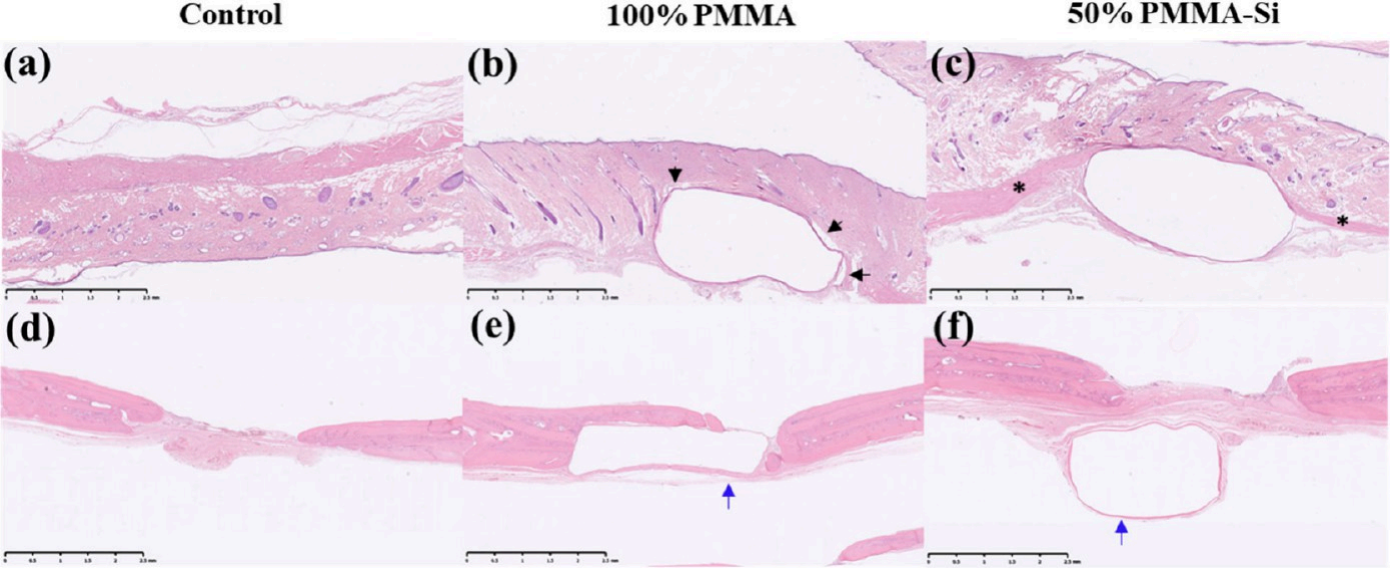
H&E-stained sections of scaffolds implanted in two different locations. The first row shows subcutaneous implantation, while the second row represents calvarial defect implantation. (a, d) Control group, (b, e) 100% PMMA scaffold, and (c, f) 50% PMMA–Si scaffold (Scale bar: 2.5 mm).

A critical size defect (CSD) is defined as the smallest bone defect that is incapable of spontaneous healing within the physiological lifespan of the animal model. In rat models, defects in the range of 5.0 mm to 8.0 mm are generally considered critical. Specifically, a 5.0 mm defect is frequently adopted as the standard for calvarial bone models, providing a consistent and reproducible framework for evaluating bone regeneration and the efficacy of therapeutic interventions.^57,58^ To evaluate the bone regeneration potential of the fabricated scaffolds, an *in vivo* study was conducted using a critical-sized calvarial defect model in SD rats with diameter of 5 mm, were surgically created in the skull under sterile conditions. The left defect was implanted with a 100% PMMA scaffold, while the right defect was implanted with scaffold 50% PMMA–Si, as shown in Figure 7a. Figure 7b–k displays the micro-CT scan results of calvarial defects in SD rats at 1, 2, 4, 8, and 12 weeks, depicting the progression of bone healing. Table 4 provides a detailed summary of area of new bone formation (%) the throughout the study period. The micro-CT analysis reveals a significant improvement in bone regeneration with both 100% PMMA and 50% PMMA–Si scaffolds compared to the control group over 12 weeks. The 50% PMMA–Si scaffold consistently showed the greatest bone formation, culminating in a new bone growth area of 63.34 ± 1.18% at Week 12, significantly outperforming both the 100% PMMA (52.45 ± 1.32%) and control (43.246 ± 2.12%). During the initial time points (Weeks 1 and 2), new bone formation was limited across all groups. However, both scaffold groups demonstrated superior osteoconductive properties relative to the control, with the 50% PMMA–Si scaffold showing slightly better bone growth than 100% PMMA. By Week 4, the difference became more pronounced, with the 50% PMMA–Si group achieving a bone growth area of 46.06 ± 3.28%, compared to 37.53 ± 1.82% for 100% PMMA and 30.90 ± 1.51% for the control group. This trend continued at Week 8, where the 50% PMMA–Si scaffold reached 55.31 ± 0.43%, reflecting enhanced osteoinductive potential, likely due to the bioactivity of silicon ions promoting osteoblast differentiation and mineralization. At Week 12, the significant increase in new bone growth for the 50% PMMA–Si group indicates that silicon incorporation accelerates bone remodeling and regeneration. The 100% PMMA scaffold also showed substantial bone growth, but the lower performance compared to 50% PMMA–Si suggests that pure PMMA may lack the bioactive cues provided by silicon, which is critical in promoting faster bone healing.

**Figure 7:**
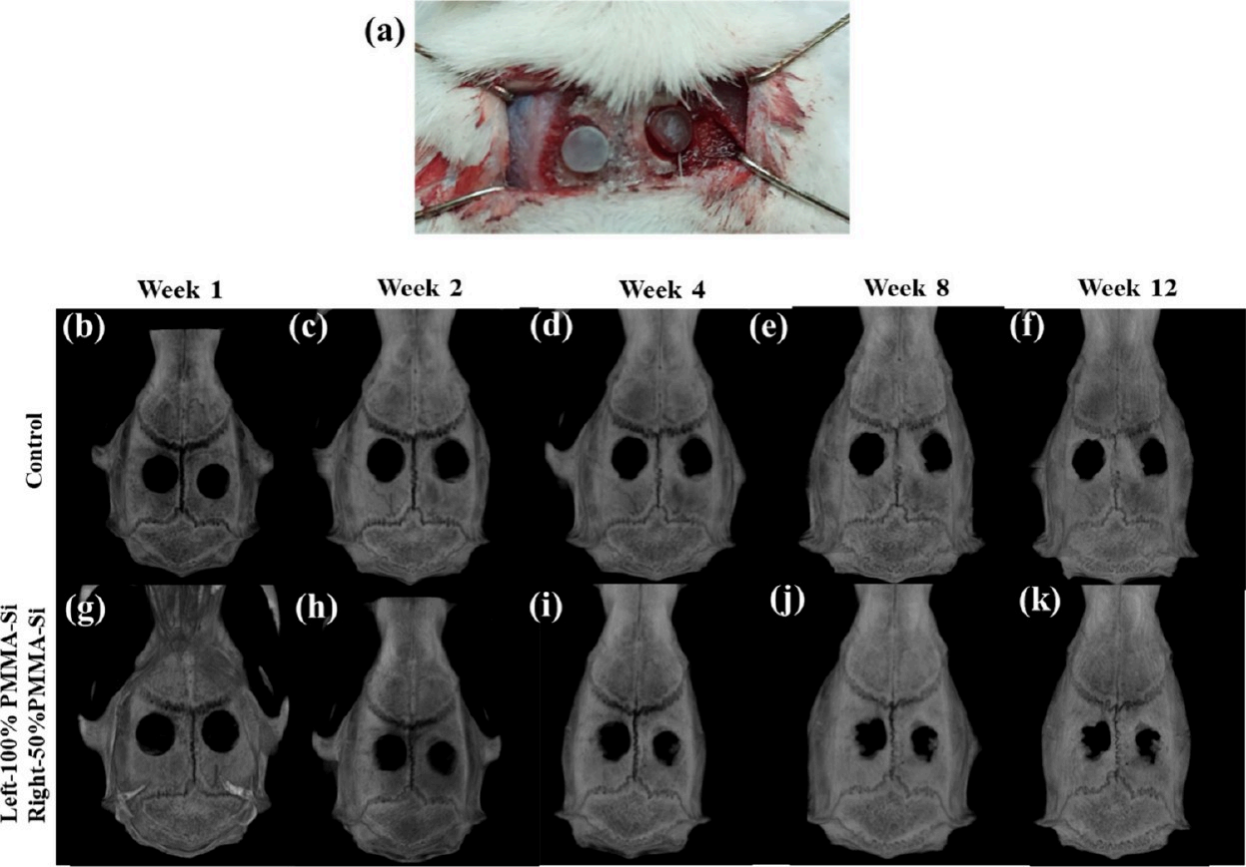
(a) *In vivo* implantation of scaffolds in a critical-sized calvarial defect (5 mm) model in a SD rat. (b–k) Micro-CT images showing calvarial bone healing in control and experimental groups over a 12-week period. The images are organized by time points at weeks 1, 2, 4, 8, and 12. The first row (b–f) represents the control group, while the second row (g–k) depicts samples treated with 100% PMMA (pure PMMA) on the left defect and 50% PMMA–Si on the right defect.

**Table 4:** Areas of New Bone Formation (%) in Control, 100% PMMA, and 50% PMMA–Si Groups over 12 Weeks^a^

week	control	100% PMMA	50% PMMA–Si
week 1	2.68 ± 1.20%	4.56 ± 1.44%	3.95 ± 0.97%
week 2	6.60 ± 2.53%	8.18 ± 1.09%	8.69 ± 2.16%
week 4	30.90 ± 1.51%	37.53 ± 1.82%**	46.06 ± 3.28%**
week 8	38.97 ± 3.33%	48.76 ± 0.62%*	55.31 ± 0.43%**
week 12	43.246 ± 2.12%	52.45 ± 1.32%**	63.34 ± 1.18%***

a Results were obtained using ImageJ software and are presented as mean ± SD (*n* = 3 for each group). The * symbols indicate statistical significance for control vs other groups (*p < 0.05, **p < 0.01, ***p < 0.001).

The histological images across control, 100% PMMA, and 50% PMMA–Si groups illustrate the progressive stages of new bone formation in calvarial defects over 12 weeks. H&E and MT staining were used to track new bone tissue regeneration and integration (Figures 8 and 9). During the initial weeks (1 and 2), bone growth was minimal in the control group, whereas more significant bone development was observed in the 100% PMMA and 50% PMMA–Si groups. This limited growth in the control group likely reflects the absence of osteoconductive materials, which support early bone formation. In contrast, the 100% PMMA and 50% PMMA–Si groups demonstrated increased early bone formation because of the ability of the material that promote cell attachment and tissue response. By weeks 4, 8, and 12, although bone growth in the control group increased, it remained lower than in the 100% PMMA and 50% PMMA–Si groups, which continued to support a more robust tissue response. The 50% PMMA–Si group, in particular, demonstrated the greatest amount of new bone formation. The enhanced growth in this group can be attributed to the incorporation of Si, which has been shown to stimulate osteoblast activity and improve mineral deposition, thereby accelerating bone regeneration.^59^ Furthermore, by weeks 12 and 18, new bone formation was predominantly concentrated around the outer edges of the implants.

**Figure 8:**
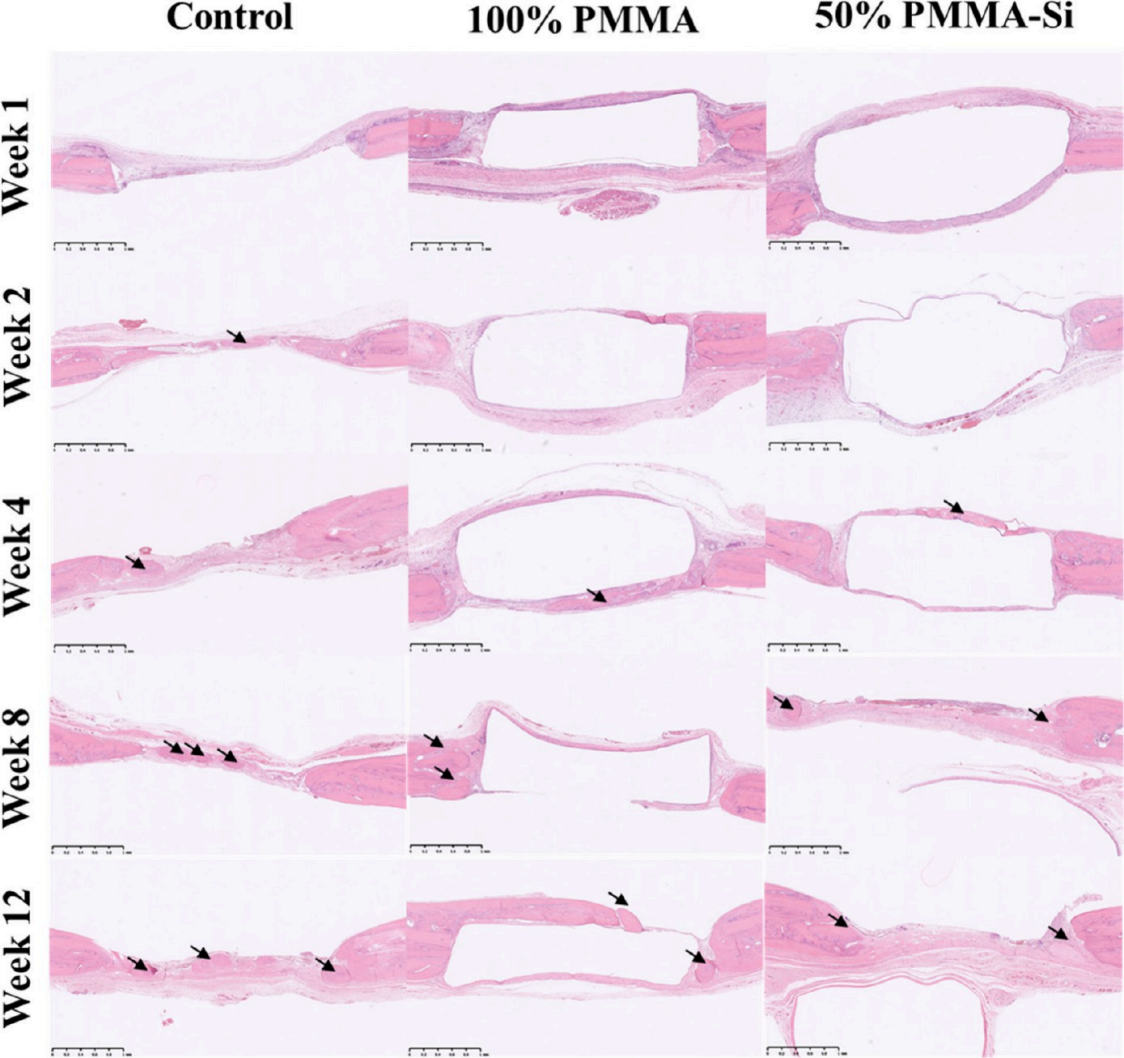
Histological analysis of bone regeneration in the rat calvarial defect model for control, 100% PMMA, and 50% PMMA–Si groups at weeks 1, 2, 4, 8, and 12, stained with H&E. The black arrow indicates new bone formation. Scale bars: 1 mm).

**Figure 9:**
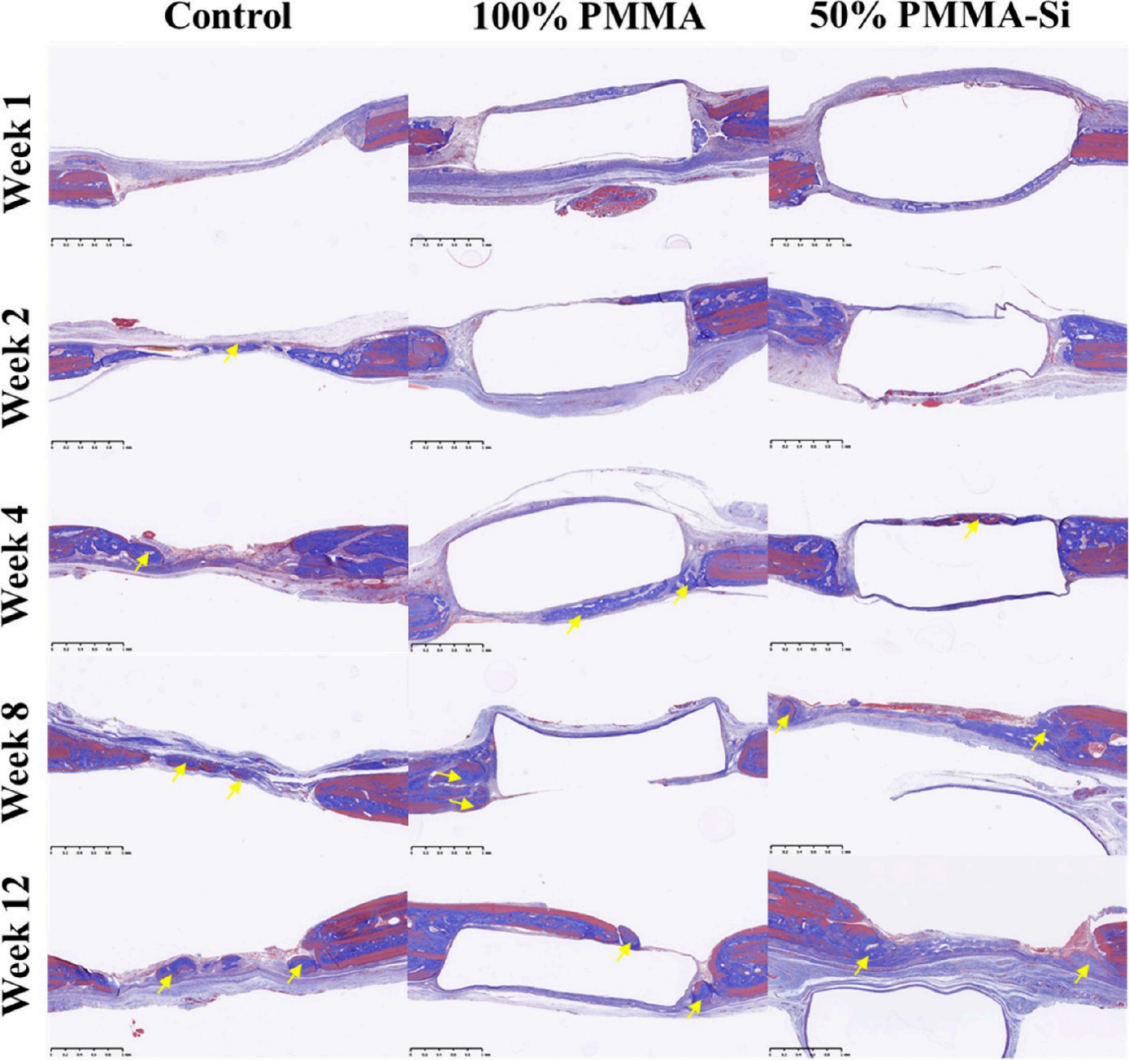
Histological analysis of bone regeneration in the rat calvarial defect model for control, 100% PMMA, and 50% PMMA–Si groups at weeks 1, 2, 4, 8, and 12, stained with MT staining. The yellow arrow indicates new bone formation. Scale bars: 1 mm).

In MT staining, during 8 and 12 weeks of control group, blue staining is still prominent, with minimal red, indicating that while collagen is present, full mineralization and maturation of the bone matrix have not occurred. In 100% PMMA and 50% PMMA–Si, high levels of blue staining, with scattered red, suggest that although there is an increase in collagen content, the matrix is still largely unmineralized. The presence of red in some areas indicates the beginning stages of mineralization. Silicon (Si) has been shown to enhance bone formation by promoting cell proliferation and upregulating bone-related genes, such as collagen type I, bone morphogenetic protein-2 (BMP-2), and runt-related transcription factor 2 (Runx-2), through activation of the extracellular signal-regulated kinase (ERK) pathway. In addition to these effects, Si plays a role in the early stages of biomineralization, suppresses osteoclast activity, and increases the production of osteoprotegerin (OPG), which counteracts the catabolic effects of receptor activator of nuclear factor κB ligand (RANKL).^60^ Furthermore, Si supports osteogenesis through the activation of key signaling pathways, including the mitogen-activated protein kinase (MAPK)-ERK and phosphoinositide 3-kinase (PI3K)-Akt-mammalian target of rapamycin (mTOR) pathways. Si also modulates cellular responses in bone marrow stromal cells (BMSCs) via WNT and sonic hedgehog (SHH) signaling pathways.^61,62^

## 4 Conclusions

In this work, we developed a PMMA–Si hybrid scaffold aimed at overcoming the inherent limitations of PMMA in BTE by enhancing its bioactivity and osseointegration potential. Utilizing TEOS as the silica source and GPTMS as a coupling agent, silica was successfully integrated into the PMMA matrix to improve cellular interactions and biological performance. *In vitro* evaluations demonstrated that the PMMA–Si scaffold significantly enhances cell proliferation, adhesion, and osteogenic differentiation, addressing the shortcomings of PMMA. Additionally, *in vivo* evaluation in rat calvarial defect model over 12 weeks confirmed that the PMMA–Si scaffold supports substantial bone regeneration and integration with the surrounding tissue. These results indicate that the PMMA–Si hybrid scaffold combines favorable mechanical stability with enhanced bioactivity, offering a promising solution for bone regeneration applications. A limitation of this study is that the scaffold material is nondegradable, which may restrict its applicability in cases where complete scaffold resorption and replacement by native bone are desired. Nevertheless, the long-term stability of the scaffold offers sustained mechanical support and an osteoconductive environment, comparable to clinically established nondegradable materials such as titanium and PMMA, thereby maintaining its relevance for load-bearing and reconstructive applications.

## Data Availability

Data will be made available on request.
